# Advances in clinical trial design: Weaving tomorrow’s TB treatments

**DOI:** 10.1371/journal.pmed.1003059

**Published:** 2020-02-27

**Authors:** Christian Lienhardt, Andrew Nunn, Richard Chaisson, Andrew A. Vernon, Matteo Zignol, Payam Nahid, Eric Delaporte, Tereza Kasaeva

**Affiliations:** 1 Unité Mixte Internationale TransVIHMI, UMI 233 IRD–U1175 INSERM—Université de Montpellier, Institut de Recherche pour le Développement (IRD), Montpellier, France; 2 MRC Clinical Trials Unit at UCL, Institute of Clinical Trials and Methodology, University College London, London, United Kingdom; 3 Center for Tuberculosis Research, Johns Hopkins University School of Medicine, Baltimore, United States of America; 4 Division of TB Elimination, National Center for HIV, Viral Hepatitis, STD and TB Prevention, Centers for Disease Control and Prevention, Atlanta, Georgia, United States of America; 5 Global TB Programme, World Health Organization, Geneva, Switzerland; 6 UCSF Center for Tuberculosis and Division of Pulmonary and Critical Care Medicine, University of California San Francisco, San Francisco, California, United States of America

## Abstract

Christian Lienhardt and co-authors discuss the conclusions of the PLOS Medicine Collection on advances in clinical trial design for development of new tuberculosis treatments.

**Summary points**

- The development process for new tuberculosis (TB) regimens remains slow and costly. In this concluding paper of the *PLOS Medicine* Special Collection, we highlight the key suggestions made at a WHO Technical Consultation on “Advances in Clinical Trial Design for Development of New TB Treatments” held in 2018 to address this challenge.
- Pharmacokinetics and pharmacodynamics (PK-PD) properties of candidate drugs are critical for constructing effective combination regimens. Bridging PK-PD methods to the analysis of Phase II studies and integrating longitudinal culture results would help with clarifying dose–response relationships and to link drug exposure to bactericidal activity; this would provide valuable insights for the identification of the components of suitable regimens.
- New adaptive designs can accelerate Phase II and III trials and improve our ability to select regimens early for further investigation. Among these, the integration of extended posttreatment follow-up with collection of real-time treatment outcomes in the new hybrid Phase IIC design, with features drawn from both Phase II and Phase III trials, permits earlier identification of candidate regimens likely to succeed in Phase III.
- Once the efficacy of a regimen is demonstrated in high-quality randomized controlled trial(s), the assessment of its effectiveness under programmatic conditions may permit an estimation of the amount of nonadherence that could substantially alter the likelihood of treatment success.
- Vulnerable populations, such as children, pregnant women, and people with HIV infection, should be included in clinical trials from the outset, as these groups have unique characteristics regarding PK, safety, and efficacy, which necessitate special attention in drug and regimen development.
- We advocate here for a better systematization and harmonization of the approaches taken internationally to ensure that best practices and novel research designs are used to accelerate development of new TB regimens. By using all the creative approaches described in this Special Collection, we hope that the next generation of TB trials will bring the high-quality evidence for novel TB regimens that is required to meet the needs of the millions of new TB patients who become ill each year.

## Introduction

In Homer’s Odyssey, each night, Penelope unwove the tapestries she made in the day to delay her marriage to one of the contenders for Ulysses’ throne while awaiting his return. In tuberculosis (TB) therapeutics, major advances 40 to 50 years ago established the current 6-month short-course chemotherapy (SCC) regimen that revolutionized TB treatment—but could we have made greater progress since [1,2]? Indeed, one can wonder if, over the past 40 years, like Penelope, the TB community has been weaving novel treatment regimens out of new and repurposed drugs, then unweaving them because of negative results and an unstructured strategy for advancing the field, despite an increasingly rich pipeline [3,4]. With new opportunities to test novel combinations to shorten TB treatment, a broad reflection on the way TB trials have been carried out over the past 40 years is legitimate, and in view of the many advances in microbiology, immunology, genetics, and pharmacology, it can help us draw from the lessons learnt to weave better TB treatments for tomorrow [5].

The most serious challenge we face in developing new TB therapeutics is our inability to identify optimal regimens early and efficiently. Limitations include the lack of direct measures of treatment response, unsatisfactory surrogate endpoints of treatment effects, and the lack of reliable predictors for Phase III clinical outcomes [6]. Identifying the optimal drug combinations and the most parsimonious trial designs to evaluate them requires critical insights incorporating recent developments in pharmacology, microbiology, biomarkers, and diagnostic assays. Given the long duration and high costs of medical development, and in view of the limited funding for TB research and development [7], it is crucially important to reassess the best practices for the development of the new TB treatment regimens of the future. In the current paper, we offer an assessment of challenges and dogma addressed by the WHO Technical Consultation on “Advances in Clinical Trial Design for Development of New TB Treatments” that took place in Glion-sur-Montreux, Switzerland, March 14–16, 2018 [8].

### 1. How can we bridge preclinical data into clinical trials and identify the pharmacokinetics and pharmacodynamics parameters that correlate best with bactericidal efficacy and toxicity in vivo?

The foundation of SCC rests on the evidence that complete sterilization of tuberculous lesions in the lungs requires at least 6 months of treatment [9,10] because of the presence of slow-growing or nonmultiplying bacilli, termed “persisters” [11]. The evidence is that these persisters are heterogeneous in nature, and their mechanism of formation results from multiple pathways [12]. Thus, although antibiotics are classically developed based on their activity against actively growing bacteria, drugs that kill the slowly or nonreplicating bacilli, like rifampicin, are critically important to shorten TB chemotherapy while retaining high efficacy [13].

Despite promising data from mouse models [14] and human studies of 2-month culture conversion rates [15,16] suggesting a potential for treatment shortening, 3 independent Phase III trials of fluoroquinolone-containing regimens for drug-susceptible tuberculosis failed to show efficacy in reducing the duration of treatment to 4 months, suggesting that treatment shortening may not be feasible with the current drugs—or only in patients with limited risk factors and paucibacillary disease [17]. So, how can we ensure that new regimens with preclinical and in vitro promise will translate into sterilizing efficacy in humans?

In TB drug development, pharmacokinetics and pharmacodynamics (PK-PD) studies are generally carried out to assess the relationship between the blood and tissue levels of a new compound and the plasma or serum bactericidal activity of the compound against *Mycobacterium tuberculosis*. PK-PD modeling is now a routine component of preclinical studies [18]. PK-PD relationships are typically evaluated based on drug exposures in plasma, but this fails to consider the varying exposure of bacterial populations in diverse lesion compartments [19,20]. The ability of drugs to penetrate anatomic lesions and to kill both active and quiescent bacilli should be considered early in the drug development process, informing the rational combination of drugs with complementary activity against the bacterial subpopulations present in the lesions [21].

Identifying the ideal synergistic use of bacteriostatic (i.e., growth inhibiting) and bactericidal antimycobacterial agents in combination as well as the timing and duration of their use across treatment phases remains a significant challenge in TB therapeutics. Mechanistic models and tools for regimen and dose optimization that evaluate the lesion-focused time course of drug levels following various drug combinations, doses, and schedules have been developed, which may lead to improved regimen selection [22]. Recently, artificial-intelligence-enabled parabolic response surface (AI-PRS) used in combination with in vitro high-throughput models has been proposed for identifying synergistic drugs to treat TB [23]. Additional complementary in vivo and clinical trials data are needed to determine whether these newer model-based techniques can facilitate the identification of maximally potent, safe, and tolerable shorter course regimens of the future.

Translational quantitative pharmacologic modeling provides an opportunity to identify preclinical and clinical PK-PD parameters that correlate best with bactericidal efficacy and toxicity [24] and to assess sputum culture results in early phase trials with clinically relevant endpoints in later-phase trials [25]. Learning from preclinical PK-PD properties of candidate drugs is critical for constructing effective combination regimens and providing an understanding of the contribution of specific agents to the entire regimen. Integrating microbiologic determinants, such as minimum inhibitory concentrations, with quantitative longitudinal culture results and PK-PD assessments should yield valuable insights during all phases of drug development. These quantitative PK-PD approaches will guide optimal drug dosing, as well as inform the assessment of drug–drug interactions [26]. This argues for the development of a standardized preclinical/clinical translational PK-PD modeling strategy for TB drug combinations with robust predictive features to guide rational selection of regimens to be moved forward into clinical development, support the selection of dose ranges to be studied, and provide quantitative predictions of clinical trial outcomes [27,28].

Based on this, the WHO Technical Consultation suggested that Phase II and Phase III trials systematically include PK sampling, so that PK-PD assessments linking drug exposures to bactericidal activity and treatment outcomes can be performed. Such analyses should account for other factors likely to affect outcomes, including disease severity and treatment adherence (Table 1) [8].

**Table 1:** Key gaps identified in the WHO Technical Consultation and suggested solutions [8].

Identified gaps	Proposed solutions	Additional comments
How can we bridge preclinical data into clinical trials and identify the PK-PD measurements that correlate best with bactericidal efficacy and toxicity in vivo?	Phase II and Phase III trials provide opportunities to collect rich and informative data on drug exposures and microbiological response over time. These trials should all include PK sampling, so that PK-PD assessments, linking drug exposures to bactericidal activity and ultimate treatment outcomes, can be performed; these analyses should account for other factors likely to affect outcomes, including disease severity and treatment adherence.	Development and validation of novel biomarkers should be integrated in all PK-PD activities to allow for rapid assessment of the biomarkers and properties of future potential surrogate for bacterial load.
Do we have the best tools currently to identify relevant drug combinations to transition from Phase II to Phase III trials?	Phase IIB/C studies, with arms testing different doses and duration and with collection of treatment outcomes, are likely to strengthen the process for identifying candidate regimens likely to succeed in Phase III.	More quantitative, longitudinal, and time-to-event measures (time-to-positivity on liquid media, time-to-stable culture conversion) are now in common use and are endorsed for broad uptake as viable alternatives to single time-point dichotomous endpoints. Adaptive approaches offer potential reductions in sample size.
How can we overcome the long duration, cost, and constraints of Phase III trials and simplify them without hampering validity and wider drug development?	Both noninferiority and superiority designs are relevant for studies of new TB regimens; their use depends on the indication (drug-susceptible or drug-resistant TB) and on the intended use and value proposition of the new regimen—e.g., better efficacy or shorter duration. New adaptive designs can accelerate Phase II and III trials and improve our ability to select regimens for further investigation.	Innovative, efficient designs (e.g., adaptive strategy designs) need to be further explored for TB drug and regimen development. Many have the potential to accelerate and enhance ability to learn.
What is the role of treatment adherence in development of new TB therapeutics?	Adherence remains an under-valued but important determinant of treatment success. More attention to this domain can help to address the global challenge of treatment default. High-quality data are needed to establish the efficacy and reliability of new methods to measure and sustain adherence.	Ensuring and measuring adherence in clinical trials are essential to correctly interpret results of the trials. Both explanatory and pragmatic trials are needed to answer questions about efficacy and safety and about expected effectiveness in programmatic conditions that includes assessment of adherence.
How can we include key populations, such as children, pregnant women, and people with HIV infection, in clinical trials from the outset, rather than as an afterthought?	More attention is needed to assure the provision of evidence relevant to key subgroups, including pregnant and breastfeeding women, young children, and persons with critical comorbidities such as HIV infection. Novel designs and approaches to integrated substudies would be useful.	The limited evidence base for the prevention and treatment of TB in pregnant women should be emphasized. More PK studies of first-line, second-line, and new anti-TB drugs in pregnant women are needed. Appropriate formulations of drugs for infants and young children should be developed during the early phases of regimen development and testing, whenever feasible. Drug–drug interaction studies between anti-TB and ARV drugs should be conducted as early as feasible within regimen development. Careful joint management of HIV and TB care is essential. In accordance with WHO guidelines, ART should be initiated as soon as possible for all HIV-infected participants with TB in clinical trials, and definitely within the first 8 weeks of TB treatment.

ART, antiretroviral therapy; ARV, antiretrovirals; PK-PD, pharmacokinetics and pharmacodynamics; TB, tuberculosis.

### 2. Do we have the best tools currently to identify relevant drug combinations to transition from Phase II to Phase III trials?

**Fig 1:**
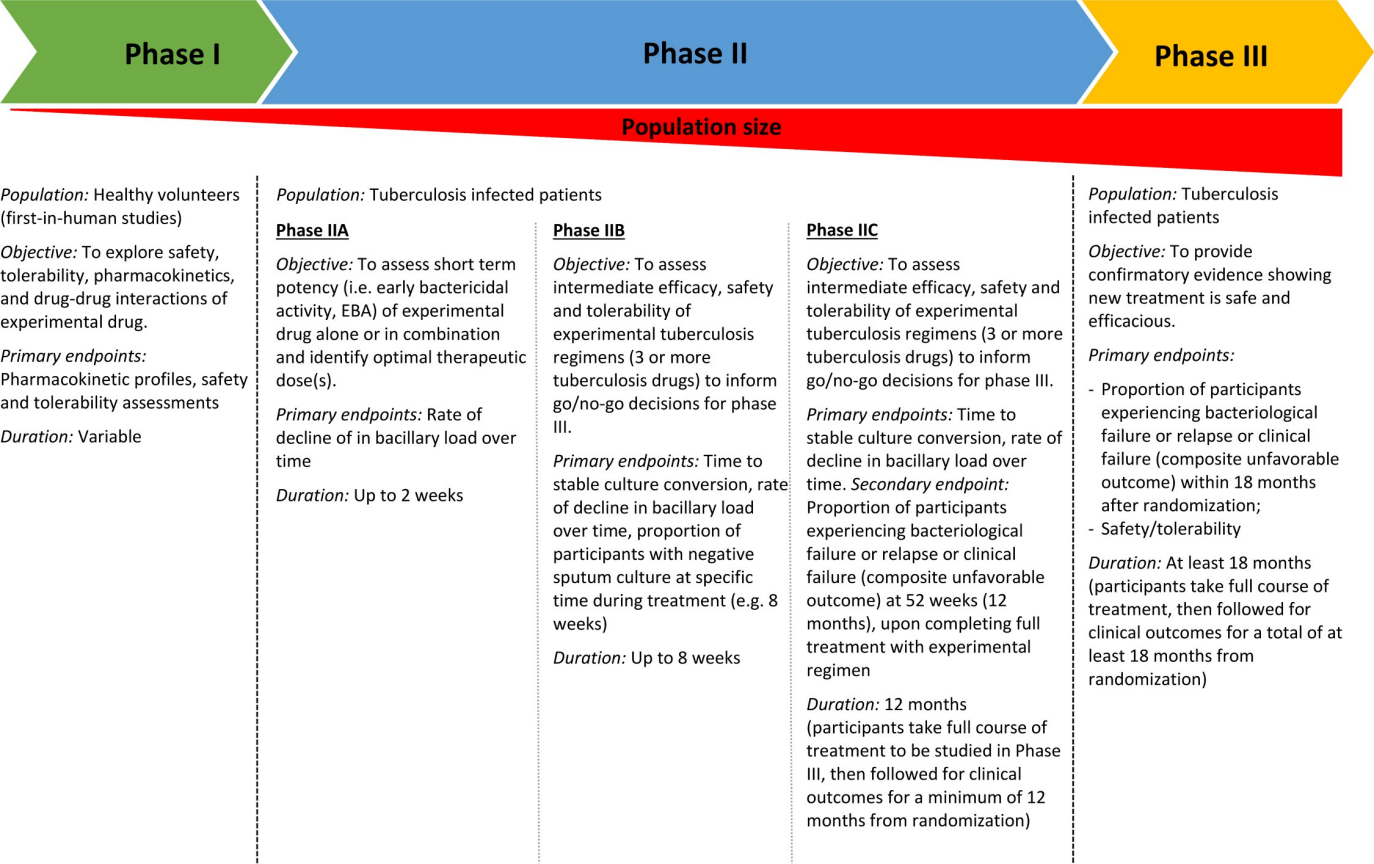
The successive clinical trial phases in human development for TB drugs/regimens [8]. TB, tuberculosis.

The early stages of clinical development should identify those drug combinations with the best safety, efficacy, and treatment duration profiles to bring to Phase III trials [29]. Phase IIA studies assist in dose-finding and provide early evidence of antibacterial activity (**Fig 1**). Phase IIB studies test more stringently the efficacy of new drug regimens, using generally sputum culture conversion at 8 weeks as an endpoint [30]. Unhappily, as shown with the fluoroquinolone trials, Phase IIB studies are insufficient for predicting long-term outcomes and may fail to identify the degree of improved culture conversion necessary to achieve substantial treatment shortening [31].

Innovations in recent years have enhanced the information gathered in these studies and have streamlined the selection of regimens. First, the application of PK-PD methods to the analysis of Phase IIA studies clarifies dose–response relationships and reinforces the validity of a shift of focus from single drugs to combinations of drugs [32,33]. New approaches to Phase IIB studies have been proposed, based on intensive sampling of sputum at various time points, with longitudinal statistical modeling of quantitative bacteriology, time-to-positivity in mycobacterial growth indicator tube (MGIT) system, or time-to-culture conversion data [34,35]. Because these outcomes are measured on a continuous rather than a binary scale, they are more sensitive to differences than the traditional 8-week endpoint.

Two new approaches have been proposed to enhance the capacity for early selection of relevant combinations to bring from Phase II to Phase III testing. The “multi-arm multi-stage” (MAMS) design allows testing of a broad range of combinations and dose levels without requiring a large sample size [36]. The second is a hybrid approach combining Phase II and Phase III features, the “Selection Trial with Extended Post-treatment follow-up” (STEP) Phase IIC, wherein limited long-term follow-up data on relapse are collected as well as data on culture conversion; this permits estimation of a Bayesian prediction interval for the likely results of a future Phase III trial [37]. Such Phase IIB/C studies, with arms testing different doses and duration and with collection of treatment outcomes, are likely to strengthen the process for identifying candidate regimens likely to succeed in Phase III [8] (**Table 1**).

### 3. How can we overcome the long duration, cost, and constraints of Phase III trials and simplify them without hampering validity and wider drug development?

Phase III confirmatory trials of TB treatment are high-cost undertakings, requiring large numbers of patients followed for long periods of time [38]. Innovative Phase III trial designs are needed for more efficient evaluation of a greater number of regimens with fewer patients and fewer resources, ensuring delivery of high-quality evidence for well-informed decisions by regulators, policy makers, healthcare providers, and patients.

Superiority trials provide robust evidence of benefit from a new drug or regimen when compared with a suboptimal standard of care. Developing new regimens for the treatment of drug-susceptible TB is, however, challenging because of the high cure rates achieved with current standard SCC under trial conditions. Noninferiority designs are more appropriate when new regimens may have practical advantages over current standard therapy (e.g., being shorter in duration or easier to adhere to) and thus may be preferred in real-life settings when such benefits may be advantageous even if the tested intervention is modestly less efficacious [39]. This margin of acceptance is defined by the noninferiority margin or delta. How narrow or wide this margin should be and how this translates into acceptable losses and desired gains is a matter of debate. A novel method is proposed that weighs potential gains and losses with the new regimen, which can then be translated into numbers of patients who would either benefit from, or be harmed with, the test intervention [40]. To minimize the possibility of biocreep (that occurs in noninferiority trials when a slightly inferior treatment is considered effective and becomes the active control in subsequent trials), the best available treatment should always be used as the control, and the margin of noninferiority should be determined on the estimated effect of the control, accounting for the variability and likely sources of bias in the estimate [41].

If there is a choice, superiority trials will always be preferred to noninferiority trials, whether in drug susceptible or drug resistant TB. It is better to be able to conclude that a new intervention is significantly better than standard treatment than that the new treatment is as good within certain limits, which is less persuasive in terms of benefit and subject to the somewhat arbitrary choice of noninferiority margins. Noninferiority becomes the design of choice when the control regimen is likely to perform to a very high level of success, a setting in which exceptionally large numbers would be required to demonstrate significant benefit. This situation is more likely to pertain in drug susceptible TB than in drug resistant TB, which, in most settings, has suboptimal results. A noninferiority design was used in the STREAM Stage 1 trial in which the proposed intervention was of substantially shorter duration, which, if demonstrated, would be of considerable benefit to patients and health services, irrespective of whether it was found to be noninferior or superior to the long-duration control [42]. Future trials in drug resistant TB in the next few years may be either of superiority or noninferiority design depending on assumptions regarding the control regimen and the benefits expected from the intervention regimen(s).

Adaptive strategies, using the MAMS design [43] or employing adjusted randomization so that more patients are enrolled into the more promising arms (so-called Bayesian adaptive randomization) [44], can improve flexibility in trial conduct by allowing changes to defined features after the trial has begun, provided that these potential changes are prespecified in the protocol.

In short, both noninferiority and superiority designs are relevant for studies of new TB regimens, depending on the indication (drug-susceptible or drug-resistant TB) and on the intended use of the new regimen, as well as on sample size considerations. New adaptive designs can accelerate Phase II and III trials and improve our ability to select regimens for further investigation. (**Table 1**).

### 4. What is the role of treatment adherence in development of new TB therapeutics?

In a meta-analysis of the trials of fluoroquinolones-containing 4-month regimens for drug-susceptible TB, modest nonadherence was associated with significantly increased risk of unfavorable outcome, in both experimental and control regimens [17]. This underscores the importance of the quality of execution in noninferiority trials, as differential adherence across treatment arms could lead to erroneous conclusions about treatment efficacy [39,45].

To evaluate how adherence influences outcomes for specific regimens, per-protocol analyses should assess a range of thresholds for acceptable adherence (e.g., 95%, 90%, 80%) [46]. A stronger analytic approach might evaluate the effect of baseline prerandomization variables associated with poor adherence on trial outcomes [47]. Defining “significant” nonadherence is difficult; it depends on multiple factors specific to each trial, including the PK of the individual drugs, the dosing schedule, and other risk factors and comorbidities that could influence the risk of treatment failure/relapse. Once the efficacy of a regimen is demonstrated in controlled trials, the assessment of its effectiveness under conditions close to programmatic reality, for instance, through the conduct of observational studies or pragmatic trials [48], could permit estimation of the amount of nonadherence that would substantively alter the likelihood of treatment success. Such an approach was applied in trials assessing various methods of directly observed therapy, as well as in treatment of DR-TB [49–51].

From the above, it appears that adherence remains an under-valued but important determinant of treatment success. Therefore, within clinical trials, it is necessary to measure adherence carefully in order to know the extent to which a regimen might be vulnerable to reduced adherence particularly under program conditions. More attention to this domain will help address the global challenge of treatment default. High-quality data are needed to establish the efficacy and reliability of new methods to measure and sustain adherence (**Table 1**).

### 5. How can we include vulnerable populations, such as children, pregnant women, and people with HIV infection in clinical trials from the outset, rather than as an afterthought?

Populations such as pregnant or breastfeeding women and very young children have been excluded from trials (or at best, grossly under-represented) because of the potential risks of new drugs. These key populations, together with HIV-infected patients, form a substantial proportion of the global TB burden and have unique characteristics regarding PK, safety, and efficacy, which necessitate special attention in drug and regimen development [52].

Concerns of potential harm from TB therapeutics to mother and fetus have led to exclusion of pregnant women from most trials of TB therapies [53]. As a result, evidence for TB treatment during pregnancy or breastfeeding has come mainly from case reports and small series [54]. Including pregnant women in TB trials would provide more rigorous evidence of safety and activity than post-marketing surveillance [55]. TB trials should include experts in maternal-fetal medicine and the care of pregnant women who can determine reasonable approaches for risk/benefit assessment in this population.

Children account for approximately 10% of all TB cases, and the effects of age and weight on drugs’ PK are most pronounced and challenging to predict in this population. Inclusion of children in TB drug development requires specific attention to trial design, including the definition of trial outcomes, timing of inclusion, and ethical considerations [56]. The inclusion of children (or development of integrated substudies) must be carefully considered and encouraged for new TB regimens [52].

The care of HIV-infected TB patients and the optimal use and timing of ART during TB treatment has dramatically evolved in recent years [57]. Treatment outcome in HIV-infected patients is highly influenced by proper management of ART, including a recognition of potential interactions between some antiretrovirals and TB drugs, particularly the rifamycins [58]. Carefully designed drug–drug interaction studies are a major element of clinical research on TB therapeutics in HIV-infected persons that should be conducted early in drug development. Within a clinical trial, provision of expert clinical management for patients with coinfection is extremely important.

We believe that more attention is needed to provide evidence relevant to important subgroups, including pregnant and breastfeeding women, young children, and persons with HIV infection (**Table 1**).

### Conclusion: Weaving better TB treatments for tomorrow

After years of neglect, more than 30 human trials are currently testing various drugs or drug combinations for the treatment of TB [59]. At least 10 of these trials investigate shorter treatments for drug-susceptible TB, and a further 10 test new combinations for shorter and less toxic treatment of drug-resistant TB. Although this renaissance in TB therapeutics research is welcome, the overall structure of the field remains an uncoordinated and fragmented effort by numerous research groups and consortia pursuing their own goals, with dependence on access to products, funding, and enrollment capacity. The current process appears less systematic than the stepwise approach taken by the British Medical Research Council (MRC) 50 years ago. With the current uncoordinated approach, are we helping to finish Penelope’s tapestry (after Ulysses’ return), or are we just unweaving it again? In this respect, it is important to note that pretomanid, a new chemical entity, has been recently approved by the US Food and Drug Administration (FDA) for the treatment of adult patients with extensively drug-resistant pulmonary TB, in combination with bedaquiline and linezolid, based on a single arm, noncontrolled, nonblinded study in 109 people [60]. Although the study achieved a major step in the treatment of this very difficult-to-treat condition, it is noteworthy that it also bypassed some of the normal requirements for randomized controlled trials of new drugs or drug combinations. This may be considered acceptable given the absence of successful standard treatment of extensively drug-resistant TB (XDR TB) and the consequent high probability of death (26%), and not randomizing patients can be reasonable when the intervention arm is likely to confer a potential benefit and when the health condition under study does not have any cure (e.g., Ebola virus disease [61]). However, for the investigation of new treatments of TB and multidrug-resistant (MDR)-TB, conditions for which a reasonable standard of care exists, the use of full Phase III randomized controlled trials should be the rule. Moreover, even though high-quality programmatic, observational data can be invaluable for understanding the performance of regimens in field conditions and for policy decision-making, such data cannot replace the need for high-quality randomized controlled trials to evaluate the efficacy, safety, and tolerability of a new treatment regimen that are key for policy development.

The articles in this *PLOS Medicine* Special Collection describe promising innovations in the search for new TB treatments. These have the potential to improve the rational identification of regimens that can be swiftly brought from early to late clinical development phases, reduce development risk, and accelerate clinical progress in TB therapy, increasing our confidence that the regimens selected for Phase III trials contain the right drugs at the right doses without deleterious drug–drug interactions. Through the use of appropriate research designs and selection of adequate endpoints, we can produce high-quality evidence for the transformation of TB treatment that is essential for the development of guidelines and ensuring strength of recommendations [62]. This requires close interaction between researchers designing the next generation of TB trials, regulators, policy makers, and advocacy groups to achieve best harmonization of the research pipeline and the subsequent policies on use and access to TB medicines [63].

The End TB Strategy calls for the introduction of new tools by 2025 in order to reach the 2030 targets of a 90% reduction in TB deaths and 80% reduction in TB incidence compared with 2015 levels [64]. Achieving these targets requires the development and introduction of new tools, in addition to ensuring universal access to existing technologies, including shorter, safer, and more effective treatment for all forms of TB. Making progress toward this goal requires maintenance of a robust pipeline of new compounds and improvements in treatment of drug-susceptible and drug-resistant TB using novel combinations of new and repurposed drugs [65].

We advocate here for the international adoption of a better harmonized approach to regimen development to ensure that best practices are used to accelerate development of new TB regimens. By using all the creative approaches described in this Collection, we hope that the next generation of TB trials will yield high-quality evidence for novel regimens that meets the needs of the 10 million persons who become ill with TB each year. Such an approach should help us to reduce these numbers more rapidly by together weaving a tapestry of highly effective, safe, and accessible TB treatments.

## Abbreviations

FDA: Food and Drug Administration
MAMS: multi-arm multi-stage
MDR TB: multidrug-resistant tuberculosis
MGIT: mycobacterial growth indicator tube
MRC: Medical Research Council
PK-PD: pharmacokinetics and pharmacodynamics
SCC: short-course chemotherapy
STEP: Selection Trial with Extended Post-treatment follow-up
TB: tuberculosis
XDR TB: extensively drug-resistant tuberculosis
